# Aspergillus tracheobronchitis with Birt-Hogg-Dubè syndrome as a rare cause of chronic cough

**DOI:** 10.1186/s12890-022-02136-2

**Published:** 2022-11-16

**Authors:** Lianrong Huang, Fang Yi, Chen Zhan, Kefang Lai

**Affiliations:** State Key Laboratory of Respiratory Disease, National Clinical Research Center for Respiratory Disease, National Center for Respiratory Medicine, Guangzhou Institute of Respiratory Health, The First Affiliated Hospital of Guangzhou Medical University, Guangzhou, Guangdong People’s Republic of China

**Keywords:** Chronic cough, Multiple lung cysts, Aspergillus tracheobronchitis, Birt-Hogg-Dubé syndrome, Case report

## Abstract

**Background:**

Aspergillus tracheobronchitis (ATB) is confined as a condition of chronic superficial infection of tracheobronchial tree. Its diagnosis is difficult due to atypical manifestations and low detective rate of Aspergillus thus far.

**Case presentation:**

Herein, we presented a 45-year-old male patient with a sole chronic productive cough for five years referred to our cough specialist clinic. Chest high-resolution computed tomography showed multiple lung cysts predominantly located in the subpleural lesions and near the mediastinum. Neither bacteria nor fungi were identified by sputum culture. However, metagenomic next-generation sequencing in sputum detected Aspergillus fumigatus DNA. The genetic testing of whole blood suggested the germline mutation of the tumor suppressor gene folliculin, supporting a diagnosis of Birt-Hogg-Dubé (BHD) syndrome. His productive cough symptom significantly improved after receiving itraconazole treatment for 2 months. After discontinuation of antifungal treatment, there was no relapse for four months follow-up. A diagnosis of ATB with BHD syndrome was eventually established in this patient.

**Conclusion:**

ATB should be considered in any patient with prolonged unexplained productive cough. Next-generation sequencing technologies may be useful to identify ATB which is uncommon and easily ignored in clinical practice.

## Background

There are few data on chronic cough and fungal infection. As early as 2009, fungi-associated chronic cough, caused by Basidiomycetous species, was firstly reported in Japan [1]. However, Aspergillus was rarely described. Aspergillus infection often occurred in immunodeficient individuals [2]. At present, emerging evidence showed that *Aspergillus* spp. could also affect immunocompetent patients with pulmonary structural abnormalities, such as bronchiectasis and cystic fibrosis [3, 4]. Aspergillus tracheobronchitis (ATB) is confined as a chronic superficial infection of the tracheobronchial tree, presenting with persistent respiratory symptoms [5]. Its atypical manifestations and low detective rate easily resulted in delayed diagnosis and misdiagnosis. Thus, it is necessary to improve clinicians’ understanding of ATB in clinical setting. We reported a case of ATB in a patient with a medical history of Birt-Hogg-Dubé (BHD) syndrome. BHD syndrome, an autosomal dominant condition, is caused by the pathogenic mutations in the tumor suppressor gene folliculin (*FLCN*) [6]. Chronic productive cough was a central feature in this patient. Here, we emphasize the challenges of diagnosing ATB due to its atypical clinical symptoms and negative culture. In addition, ATB should be considered as one of important causes of persistent productive cough, especially in patients with structural lung damage.

## Case presentation

A 45-year-old man was admitted to our hospital with a 5-year history of chronic productive cough. The cough could be triggered by cold air and eating cold food, and mostly occurred during the day. He denied fever, chills, chest pain, chest tightness, shortness of breath, acid regurgitation, heartburn, postnasal dripping, runny nose, or hemoptysis. He had been prescribed with antitussives, expectorants, inhaled corticosteroids, antibiotics or traditional Chinese medicine, with poor outcome. He had a smoking history of 4 pack-years, which he had quit one year before. Results of physical examination were unremarkable, and the lung appeared clear, with no crackles or wheezes.

On laboratory investigations, blood tests did not show abnormal values on the total and differential leukocyte count, C-reactive protein, procalcitonin and D-dimer. Serum immunoglobulins (IgG, IgA, IgM) and complement components (C3, C4) levels were almost normal. Galactomannan was normal in both serum and bronchoalveolar lavage fluid (BALF). He had an elevated eosinophil level in sputum (4%; normal value, < 2.5%). Neither bacteria nor fungi was detected by sputum culture. Pulmonary function tests revealed a normal lung ventilation function and a mild decline in diffuse capacity of the lung for carbon monoxide. A methacholine bronchial provocation test was negative.

**Fig. 1:**
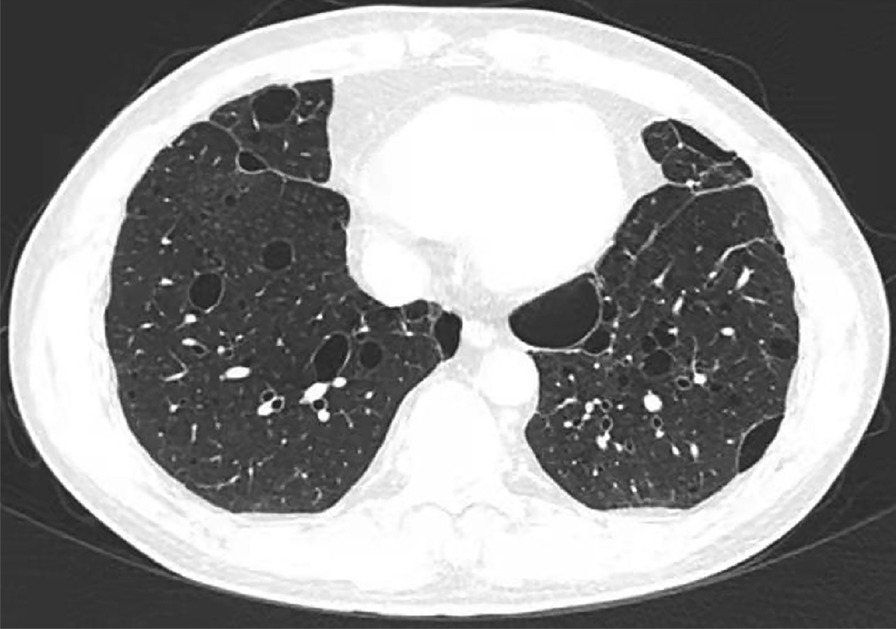
Chest high-resolution computed tomography scan showing multiple thin-walled cysts in bilateral lung and emphysematous bullae

**Fig. 2:**
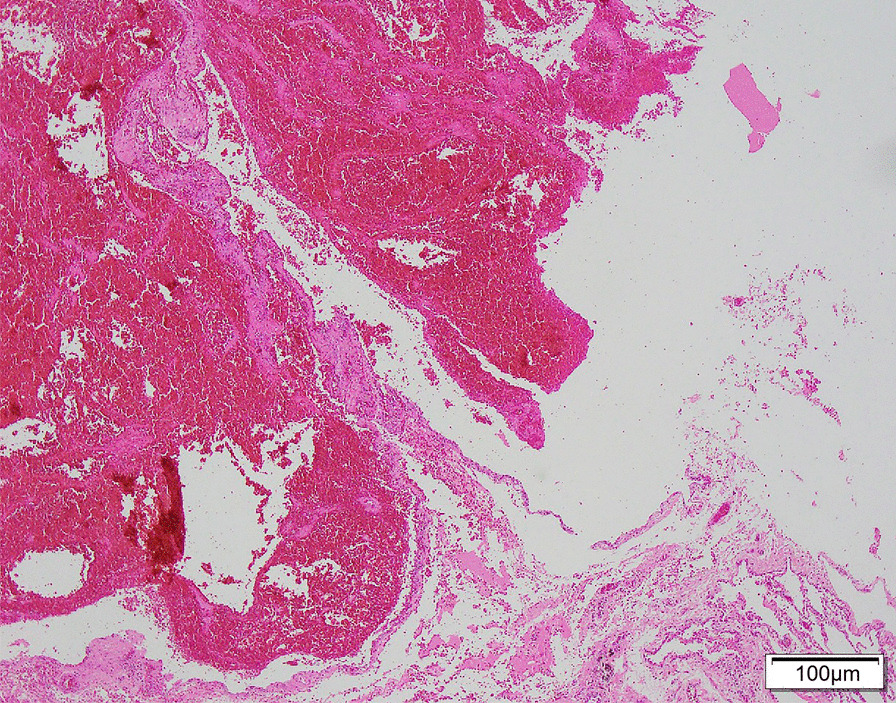
Photomicrographs of biopsy form the upper left lung obtained via video-assisted thoracoscopy with hematoxylin-eosin stain. The microscope image was visualized using an Olympus BX53 microscope at 100× magnification, and analyzed by the Logene PathQC software. This stained image shows bullous emphysema associated with pulmonary bullae

Chest high-resolution computed tomography (CT) scan was performed at least one time per year over the past five years in this patient, and showed similar results: multiple thin-walled cysts and emphysematous bullae predominantly located in the subpleural lesions and near the mediastinum (Fig. 1). No fungal ball, cavity, or aspergillomas was found. The differential diagnosis was felt to include lymphocytic interstitial pneumonia, lymphangioleiomyomatosis, pulmonary Langerhans’ cell histiocytosis, light chain deposition disease, BHD syndrome, or infectious diseases. In order to identify the etiology of productive cough and lung cysts, he underwent wedge resection of the upper left lung via video-assisted thoracoscopy. And the results of histopathology revealed bullous emphysema with pulmonary bullae (Fig. 2).

On further questioning on family history, he reported that his cousin had recurrent spontaneous pneumothorax and diffuse lung cysts. Considering family history, chest imaging and lung biopsy results in this patient, BHD syndrome appeared to be a likely cause of pulmonary cysts. He then performed the genetic test of whole blood, demonstrating the germline mutation of the tumor suppressor gene *FLCN*. Therefore, a diagnosis of BHD syndrome was made in this patient. During hospitalization, his cough and sputum production improved transiently with intravenous antibiotics and mucolytics. The etiology of cough nevertheless remained unclear. 

The symptoms worsened 3 months after hospital discharge. When he was referred to our cough specialty clinic, he complained of aggravated cough and sputum production. On auscultation, there were no crackles or wheezes on bilateral lung. Given clinical notes, previous investigations and treatment response, corticosteroid-sensitive cough (i.e., asthma, eosinophilic bronchitis and atopic cough) and upper airway cough syndrome were less likely. However, gastroesophageal reflux cough remained a likely cause of his cough. Then, a 2-weeks trial of treatment with proton pump inhibitor and promotility agents was administrated, but without any improvement for cough symptom, indicating that reflux-related cough was ruled out in this patient. Over the past several years, the patient’s cough did not relieve although a variety of antimicrobial agents had been used. However, these persistent productive cough and diffuse lung cysts still suggested a possible underlying infectious cause (i.e., fungal or virus infection). Consequently, microbiological tests were performed again in this patient. Both bacterial and fungi cultures showed negative results. Whereas, the metagenomic next-generation sequencing (mNGS) in sputum detected Aspergillus fumigatus DNA.

Given the results of mNGS, a 8-week tentative treatment with oral itraconazole (ITCZ) was prescribed in this patient. Cough visual analog scale (VAS) was used to evaluate cough severity, with a range of 0–100 mm. A reduction of cough VAS ≥ 30 mm indicated good response to treatment [7].Cough VAS of this patient significantly decreased by 70 mm after prescribing ITCZ, suggesting a diagnosis of Aspergillus infection. After discontinuation of antifungal treatment, there were no relapse and drug-related adverse events four months follow-up. A diagnosis of ATB with BHD syndrome was eventually established in this patient.

## Discussion and conclusions

Chronic cough, defined as a cough lasting for more than 8 weeks, is associated with asthma, eosinophilic bronchitis, gastroesophageal reflux disease or upper airway cough syndrome (postnasal drip) [8]. These were not the culprits in our patient, and multiple lung cysts suggested that an infectious condition may be responsible for his cough and expectoration.

In this patient, ATB was the underlying cause. ATB is a chronic superficial infection of the lower respiratory tract [5]. It is caused by Aspergillus species, ubiquitous environmental organisms. Aspergillus infection depends upon the patient’s local and systemic immune status and alterations in host tissues [5]. It is a rare disease, accounting for 5% in lung transplantations patients [9]. There is no epidemiological data on the prevalence of ATB thus far. Accumulating evidence showed that Aspergillus affected individuals with pulmonary structural abnormalities, such as bronchiectasis or cystic fibrosis [3, 4]. Despite no obvious signs of bronchiectasis or cystic fibrosis seen in our patient, he had BHD syndrome, a rare autosomal dominant disorder with diffuse lung cysts [10]. The impaired airway structure attenuated local defense functions and increased opportunistic infections. Whereas the normal immune function of this patient could prevent the spread of infection and substantially contributed to the localization of the disease, which was likely to be an important explanation of Aspergillus infection confined in the tracheobronchial tree, not lung. This pulmonary structural destruction might be an important risk factor for developing fungal colonization or infection.

ATB has a broad spectrum of symptoms. The common manifestations include chronic productive cough, tenacious mucus production, dyspnea, and recurrent exacerbations of pre-existing airway disease with a response incompletely to antibiotics [11]. Owing to lack of specificities of the symptoms, ATB is usually neglected by clinicians.

ATB is a rare cause of chronic cough, although cough symptom is often described in ATB patients. In our patient, a productive cough was a critical feature. In a small case series of patients who had ATB, common symptoms included productive cough and dyspnea [12]. Dyspnea is usually related to obstructive bronchial aspergillosis, especially in lung transplant patients [12]. The symptom of dyspnea is less reported from our patient, possibly ascribing to mild infection without abnormal pulmonary ventilation capacity.

In the present case, the diagnosis of Aspergillus infection was made based on identification of Aspergillus fumigatus DNA in sputum and good response to antifungal therapy. The isolation of Aspergillus fumigatus from the sterile sample is thought to be a gold standard of diagnosis [13]. Of these methods, the most reliable criterion of invasive fungal airway disease is demonstration of tissue invasion and damage caused by hyphae. However, Aspergillus hyphae was not visualized in lung or bronchi biopsy in our patient, which might be related to inappropriate circumstance for proliferation because of the relative normal immune state. Indeed, neither sputum nor BALF is an ideal tool to diagnose etiology of bronchitis. Increasing evidence showed the mNGS has a high sensitivity and specificity for identifying fungus [14]. And our case supported the use of mNGS test in indicating the occurrence of mild fungus infection in unexplained chronic cough. However, further cohort studies are required in the future. To identify the etiology of cough, this patient performed chest CT many times during the past five years. Nonetheless, repeated chest CT is not recommended while diagnosing chronic cough.

As early as 2009, Ogawa and his colleagues firstly reported a link between fungi and chronic cough [1]. They mainly focused on Basidiomycetous species. However, Aspergillus was rarely described. So far, fungi-associated chronic cough was less reported in other countries except for Japan. One possible reason is the low infective rate of fungi among the chronic coughers in real world. In addition, the limitation of traditional fungal detective approaches is another potential factor as well. Currently, mNGS technique performs well in identifying rare and difficult-to-detect pathogens from respiratory specimens [14], possibly providing new evidence for suspected fungal infection.

The present case shows the difficulty in recognizing ATB with BHD syndrome. Their rarity and non-specific clinical manifestations often result in delayed diagnosis in the clinic practice. Although ATB is uncommon, it should be considered in any patient with prolonged unexplained productive cough.

## Data Availability

The data that support this case report are available from the corresponding author on reasonable request. The sequencing data were uploaded in the National Center for Biotechnology Information (Accession number: SRR20662448).

## Abbreviations

ATB: Aspergillus tracheobronchitis.
BHD: Birt-Hogg-Dubé.
FLCN: folliculin.
BALF: bronchoalveolar lavage fluid.
CT: computed tomography.
VAS: visual analog scale.
mNGS: metagenomic next-generation sequencing.
ITCZ: itraconazole.
